# A Study of Relationships Between Mental Well-Being, Sleep Quality, Eating Behavior, and BMI: A Cross-Sectional Study Among University Students

**DOI:** 10.3390/ijerph22101465

**Published:** 2025-09-23

**Authors:** Linda Priede, Ilze Beitane, Loreta Beitane

**Affiliations:** 1 Food Institute, Latvia University of Life Sciences and Technologies, LV-3001 Jelgava, Latvia; lindapriede3@gmail.com; 2 Faculty of Medicine, Rīga Stradiņš University, LV-1007 Riga, Latvia; 058425@rsu.edu.lv

**Keywords:** DASS-42, R18V2, PSQI, depression, anxiety, stress, uncontrolled eating, cognitive restraint, emotional eating

## Abstract

The study aimed to conduct a multifaceted assessment of university students’ mental health, sleep quality, eating behavior, and BMI, and to investigate the relationships between these factors. The DASS-42 (Depression, Anxiety, and Stress Scale), PSQI (Pittsburgh Sleep Quality Index), free-factor eating questionnaire R18V2, and BMI were the research instruments used. The study involved 397 university students, 59.9% of which were of a healthy weight. The average scores obtained in the student assessment indicated mild to moderate depression, moderate anxiety, and normal to moderate stress levels. The average uncontrolled eating score was 43.72 ± 14.69, cognitive restraint score—32.86 ± 30.58, and emotional eating score—31.14 ± 29.00. BMI was found to have a weak but significant correlation with cognitive restraint and emotional eating. Sleep quality was found to have a moderately strong correlation with depression, anxiety, and stress. A weak but significant correlation was confirmed between emotional eating and depression, anxiety, and stress. The students were at high risk of depression, anxiety, and stress, which was correlated with poor sleep quality and bad eating behavior. These results will be used to develop a support program that promotes students’ mental well-being, which is essential for academic success.

## 1. Introduction

In today’s world, where physical achievements and visible success are often prioritized, mental health remains an obstacle to overall well-being. The World Health Organization defines mental health as a state of well-being in which a person can cope with the stresses of life, fulfill their potential, learn and work effectively, and contribute to society [1]. This issue has received more attention in recent years, especially since the onset of the global COVID-19 pandemic, which has had a significant impact on people’s mental health and well-being [2]. The changes brought about by the pandemic have affected students’ daily lives and the way they study. There has been a shift to distance learning, and restrictions have been imposed on social life, creating general uncertainty about the future. These factors have significantly impacted students’ ability to adapt and maintain emotional equilibrium [3], increasing stress and anxiety [4].

Furthermore, it should not be forgotten that the educational process itself is a stressor that can impact students’ mental well-being [5], and this should be taken into consideration in any discussion of student mental health. A study of well-being and mental health among students in European higher education showed that, on average, 17% of students in Europe reported mental health problems such as depression and anxiety. The highest rates were found in Sweden (29%) and Finland (23%), while Latvia had the second lowest rate—4% [6].

Maintaining mental health is vital because it influences people’s everyday choices. Studies show that stress and anxiety among students are directly linked to poor diets and unhealthy eating habits [7,8,9]. Antonopoulou et al. [10] concluded that following the Mediterranean diet was associated with a lower risk of depression among university students. A systematic review by Solomou et al. [7] confirmed that students who consume high-quality foods, such as vegetables, fruits, nuts, and fish, exhibit fewer signs of mental health disorders compared to those who consume poor-quality diet. A study conducted at Poznan University of Medical Sciences examined the relationship between eating habits, depressive symptoms, and lifestyle among Polish university students. The study concluded that BMI, depressive symptoms, and impulsivity were significantly higher among students with high levels of disordered eating compared to those with low levels [11].

Furthermore, Katsoulis et al. [12] revealed that young adults are the age group most at risk of experiencing changes in body weight, transitioning from a healthy weight to obesity. However, Moradi et al.’s [13] meta-analysis did not find an essential link between being overweight/obese and the risk of depression and anxiety among adolescents. A meta-analysis by Wang et al. [14] reached the opposite conclusion, indicating overweight/obese adolescents in China have significantly higher rates of depression and anxiety symptoms than those with a healthy weight. Similar observations were reported by Pelletier et al. [15], who found that a higher presence of overweight/obesity was associated with higher stress levels among college students.

Eating behaviors play a crucial role in developing healthy diet as they influence food choice decisions. The literature contains studies on eating behavior such as emotional eating, cognitive restraint, and uncontrolled eating, as these can negatively affect weight and health [12,16] and may be a precursor to eating disorders [17]. Emotional eating is characterized as an inappropriate response to negative emotions; cognitive restraint is the conscious desire to lose weight by restricting food intake; and uncontrolled eating is an inability to control amount and frequency of food consumed. Studies indicate that there are eating behavior issues among students, particularly women [11,18].

Sleep quality is a multifaceted concept that encompasses several essential factors that determine sleep efficiency and its impact on human well-being [19]. Poor sleep quality, such as short sleep duration or frequent awakenings, is a significant risk factor for mental health issues like anxiety and depression, as well as other cognitive disorders [20]. Furthermore, Kyle & Henry [21] suggested that sleep quality has a greater impact on mental health than physical health. Similar findings were reported by Clement-Carbonell et al. [22] in their study of university students, which observed a strong correlation between sleep quality and mental health. In their study, Lee et al. [23] highlighted the existing correlation between sleep quality and quality of life. They discovered that individuals with consistently good sleep quality had significantly lower stress levels and higher self-esteem compared to those with consistently poor sleep quality. A study by Pham et al. [24] of university students revealed that using electronic devices before bedtime negatively impacts sleep quality. An interesting finding emerged from a study of medical students: those with poor sleep quality achieved higher academic results, despite poor sleep quality being significantly associated with depression, anxiety, and stress [25].

Good sleep, healthy nutrition, and a healthy lifestyle are basic requirements for maintaining health. This, in turn, allows students to build successful careers and achieve good results in their studies. This is particularly important for students undergoing the transition from youth to adulthood, as they must prove themselves not only academically, but also professionally, as many of them work while studying. In order to compete successfully, students often reduce their sleep time [19] and neglect their diet [26], which can affect their mental and physical health in the long term [23,27]. It is therefore important to raise awareness of these issues and take various preventive measures to help students feel good. As findings from various studies have indicated distinct results, further research is necessary. This study aimed to conduct a multifaceted assessment of university students’ mental health (DASS-42), sleep quality (PSQI), eating behavior (R18V2), and BMI, and to investigate the relationships between these factors. Three research questions were raised in this study based on the research results available in the literature. 1. What is the relationship between BMI and eating behavior (uncontrolled eating, cognitive restraint, and emotional eating)? 2. What is the relationship between mental health (depression, anxiety, and stress) and eating behavior (uncontrolled eating, cognitive restraint, and emotional eating)? 3. What is the relationship between sleep quality and mental health (depression, anxiety, and stress)?

The results of the study are expected to reveal the current situation regarding students’ mental health, sleep quality, eating behavior, and BMI. These results will then be used to develop a support program to promote students’ mental well-being, which is essential for academic success.

## 2. Materials and Methods

### 2.1. Study Design and Participants

Permission for the study has been obtained from the Ethics Committee of the Food Institute at the Latvia University of Life Sciences and Technologies (protocol code 2024/2-1, dated 19 November 2024). The study took place from 21 November 2024, to 30 March 2025 at Latvia University of Life Sciences and Technologies (LBTU). LBTU is a state university of science. A total of 408 participants took part in the study, of whom 397 were deemed eligible. The exclusion criteria were not being a student of the Latvia University of Life Sciences and Technologies (LBTU).

**Figure 1:**
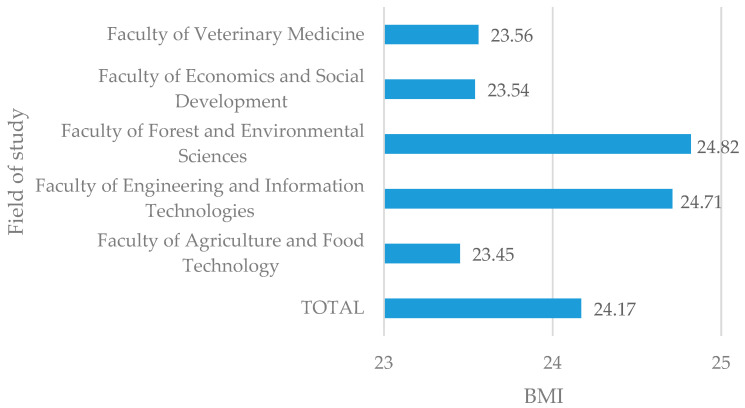
The average BMI of students depends on their field of study.

Researchers used the Google Forms tool to create an anonymous online questionnaire titled “Relationship between mental health, sleep quality, and eating habits of LBTU students”, consisting of three validated Latvian questionnaires (Figure 1). To obtain the Latvian-validated versions of questionnaires, applications had to be sent to the Psychology Laboratory at Rīga Stradiņš University. The Laboratory approved the use of the instruments within the framework of the study.

The online questionnaire was published on the social networks Instagram and Facebook. Participants were also approached via the student WhatsApp groups at LBTU, with the help of the LBTU Student Self-Government. Consent to participate in the study was considered to have been given if study participants voluntarily completed and submitted the questionnaire. At the beginning of the survey, students were informed about the study. They were told that the questionnaire was anonymous, did not include sensitive data, and could not be used to identify the research participants. All data would be used only in an aggregated form. By filling out the questionnaire, participants agreed to have their data processed as part of the study, and completing the questionnaire was considered consent to participate in the survey.

A detailed analysis was conducted based on the official annual report of LBTU, compiled as of 1 October 2024, to determine the minimum number of respondents required from university to ensure the study’s sufficient representativeness. With a 95% confidence level and a 5% margin of error, the minimum number of participants required for the study was 350 students, out of a total student population of 3880 at LBTU.

For this study, the reference criterion was set at 8.5% of the total number of students from each faculty. This means that the total number of students from each faculty is used to calculate the minimum number of respondents required for the data obtained to reflect the faculty in a partially representative way. By selecting a threshold of 8.5%, the study stuck a balance between the practicality of data collection and the need to gather sufficient information from each faculty to enable meaningful conclusions to be drawn. This approach ensures equal representation of students in different fields of study.

### 2.2. The DASS-42—The Depression, Anxiety, and Stress Scale

This is a psychological self-assessment instrument designed to measure three interrelated negative emotional states: depression, anxiety, and stress. It consists of 42 statements. The DASS-42 is a widely used, reliable instrument for assessing emotional well-being in research and clinical practice. The scale is divided into three subscales (depression, anxiety, and stress), each containing 14 questions [28]. Responses were recorded on a scale from 0 to 3, indicating the extent to which a specific experience has been relevant over the few weeks: 0—did not apply to me at all, 1—applied to me to some degree, or some of the time, 2—applied to me to a considerable degree, or a good part of time, 3—applied to me very much, or most of the time. The raw data were summed for each of the three subscales (depression, anxiety, and stress). The DASS-42, which had been adapted into Latvian by Ozolina [29], was used in the study. The Cronbach’s alpha level was 0.952 for the total DASS-42, 0.902 for the depression subscale, 0.883 for the anxiety subscale, and 0.900 for the stress subscale.

The depression, anxiety, and stress scale are designed to evaluate the severity of emotional health disorder symptoms and to identify individuals who may be at risk of depression, anxiety, or chronic stress. While it does not provide a clinical diagnosis, the scale is an effective screening tool that enables the early detection of psychological distress.

The cut-off values for depression are: normal 0–9; mild 10–13; moderate 14–20; severe 21–27; and extremely severe 28+. The cut-off values for anxiety are: normal 0–7; mild 8–9; moderate 10–14; severe 15–19; and extremely severe 20+. The cut-off values of stress are: normal 0–14; mild 15–18; moderate 19–25; severe 26–33; and extremely severe 34+.

### 2.3. Three Factors Eating Questionnaire R18V2

This is a psychological self-report instrument designed to assess an individual’s eating behavior. This method was developed from the original 51-question version (TFEQ) and modified into a shorter, more reliable 18-question version [30] that efficiently collects data in various studies. Three Factors Eating Questionnaire R18V2 was adapted into Latvian by Rasmane [31], corresponding to the original survey indicators. The Cronbach’s alpha level was found to be between 0.78 and 0.94 [30].

The 18-question scale is divided into three main subscales: uncontrolled eating, cognitive restraint, and emotional eating. Each question is rated on a four-point Likert scale: 1—definitely false, 2—mostly false, 3—mostly true, 4—definitely true. The results are converted into a scale ranging from 0 to 100 for each subscale. A higher score indicates a greater tendency towards uncontrolled eating, cognitive restraint, or emotional eating.

### 2.4. PSQI—Pittsburgh Sleep Quality Index

This is a widely used self-assessment tool in both clinical practice and scientific research. It is designed to evaluate sleep quality and disturbances experienced over the past month. It consists of 19 questions, summarized into seven components which together characterize sleep quality: subjective sleep quality, sleep duration, sleep latency, sleep efficiency, sleep disturbances, use of sleep medications, and daytime dysfunction due to sleep problems [32]. Each component is rated on a scale of 0 to 3 points: 0—not during the past month, 1—less than once a week, 2—once or twice a week, 3—three or more times a week. The total PSQI score is calculated by summing the scores of all components, with a maximum possible score of 21. A higher score indicates poorer sleep quality. The cut-off value for the results is 5 points, which is considered a clinically significant threshold for identifying individuals with sleep disorders [32].

### 2.5. BMI—Body Mass Index

The researchers calculated the BMI of students based on their reported body weight and height. The BMI was interpreted according to the WHO guidelines for adults, whereby a BMI < 18.5 is considered underweight, a BMI of 18.5–24.9 is considered a healthy weight, a BMI of 25.0–29.9 is considered overweight, and a BMI of 30.0 or higher is considered obesity [33].

### 2.6. Data Analysis

Data analysis was performed using IBM SPSS Statistics 30 and Microsoft Excel v16 programs. Descriptive statistics were calculated, including mean values, standard deviations, minimum, and maximum values. The Pearson Spearman correlation test was used to assess the relationships between BMI, PSQI, DASS-42, and R18V2. Statistical significance was determined using a *p*-value < 0.05. Normality was tested using the Kolmogorov–Smirnov test. A paired *t*-test was used to compare groups.

## 3. Results

### 3.1. Characteristics of the Study’s Participants

The study involved 397 LBTU students (Table 1), aged between 19 and 52 years, with an average age of 22.36 years. The study participants were drawn from five faculties, ensuring that each faculty was represented by at least 8.5% of its total number of students.

BMI calculations revealed that 59.9% of students were of a healthy weight, with 33.2% being categorized as overweight or obese. The average BMI among students was 24.17 (Figure 1), indicating a healthy body weight in all faculties. However, the BMI range was from 14.20 to 59.88.

An above-average BMI was observed in two faculties: the Faculty of Forest and Environmental Sciences and the Faculty of Engineering and Information Technologies.

### 3.2. The Results of DASS-42

The average scores obtained in the student assessment indicated mild to moderate depression, moderate anxiety, and the level of stress varied by faculty, ranging from normal to moderate (Table 2). Students studying veterinary medicine in the Faculty of Veterinary Medicine had the highest average scores for depression, anxiety, and stress.

In this study, the Cronbach’s alpha level was 0.960, which was close to the adapted DASS-42 scale in Latvian.

The results of the DASS-42 showed no significant differences (*p* > 0.05) in depression scores between faculties, while significantly lower anxiety scores were found in the Faculty of Forest and Environmental Sciences (*p* < 0.05) compared to the other faculties. However, stress scores revealed significant differences between faculties (*p* < 0.05), with students from the Faculty of Veterinary Medicine experiencing the highest stress levels.

**Figure 2:**
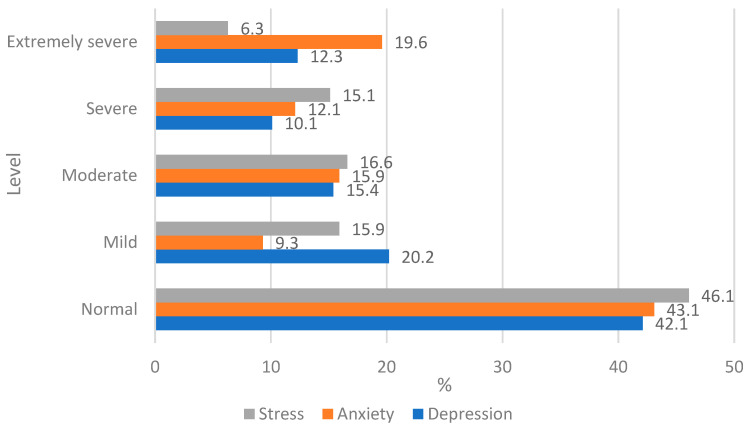
Distribution of student ratings of depression, anxiety, and stress compared to normative data.

The results of the depression, anxiety, and stress scales revealed that over 50% of students experienced emotional pressure ranging from mild to extremely severe every day (Figure 2).

Fewer than half of students know how to manage their emotions without displaying symptoms of depression, anxiety, or stress. Furthermore, 19.2% of students displayed symptoms of extremely severe anxiety, while 20.2% of students showed signs of mild depression.

### 3.3. The Results of the Three-Factors Eating Questionnaire

The average uncontrolled eating score (43.72 ± 14.69) suggests that students may struggle to control their eating habits (Table 3). This indicates possible impulsivity and difficulty regulating eating behavior. Conversely, the average cognitive restraint score (32.86 ± 30.58) suggests that some students attempt to consciously control their food intake to reduce or maintain their body weight. The average emotional eating score (31.14 ± 29.00) reveals that some students tend to eat in response to emotional factors such as stress, sadness, or boredom. This behavior may be associated with psychological stress and serve as an adaptive mechanism for emotional regulation.

No significant differences were found between students from different faculties, except for the Faculty of Economics and Social Development, where a significantly lower score for uncontrolled eating and emotional eating was determined among students (*p* < 0.05). However, students from the Faculty of Veterinary Medicine showed the highest scores in indicators such as uncontrolled eating and emotional eating.

The Cronbach’s alpha level was 0.853.

### 3.4. Pittsburgh Sleep Quality Index

According to the Pittsburgh sleep quality index, a score below 5 indicates good sleep quality. With an average score of 11.01 (Table 3), students were found to have poor sleep quality. This suggests the presence of general sleep disorders that can hurt students’ psychological and physical well-being.

PSQI results showed no significant differences in sleep quality between students across faculties (*p* > 0.05). Poor sleep quality may be associated with student life, where studying, working, and maintaining an active social life often prevent adequate sleep.

The Cronbach’s alpha level was 0.739.

### 3.5. Correlations Between BMI, PSQI, DASS-42, and R18V2

Findings on the correlation coefficient and *p*-values are outlined in Table 4.

A strong positive correlation was found between depression and anxiety, depression and stress, anxiety and stress, and uncontrolled eating and emotional eating. A moderate positive correlation was observed between PSQI and depression, PSQI and anxiety, and PSQI and stress.

## 4. Discussion

This study adopted a multidimensional approach, incorporating analysis of BMI, emotional well-being (depression, anxiety, and stress), eating habits (uncontrolled eating, cognitive restraint, and emotional eating), and sleep quality, as well as their interactions. This was one of the first studies of this kind to be conducted among students in Latvia.

This study found that nearly 60% of students were determined to be of a healthy weight. In addition, the distribution of students’ BMIs was relatively similar to that of Latvian youth (15–24 years old), with healthy BMIs found in 69.75% of cases. However, it was significantly better than the BMI distribution of the Latvian population, where healthy BMI was found in only 39.10% of cases [34]. Compared to studies of students from other countries, the prevalence of being overweight and obese among Latvian students (33.2%) was significantly higher than among Polish students (15.9%) [11], Iranian high school students (21%) [35], and Turkish students (23%) [36], but lower than among university students in the United Arab Emirates (41.1%) [37].

The DASS-21 results confirmed that, depending on the faculty, students had mild to moderate depression, moderate anxiety, and normal to moderate stress levels. Furthermore, 57.9% of students were depressed, 56.9% experienced anxiety, and 53.9% were stressed. A study of Chinese medical students found that 57.5% of them suffered from depression [38]. This was very close to the results of this study. Conversely, Zhuang et al. [39] study of Chinese students reported better results, with 30.0% prone to depression, 49.8% experiencing anxiety, and 33.9% suffering from stress. A similar result regarding depression was reported by Suwalska et al. [11] in a study of Polish students, in which 34.8% of students exhibited mild to severe depressive symptoms. In turn, a cross-sectional study in Italy found that 24% of university students were at high risk of depression [9]. This is consistent with the findings of this study, in which 22.4% of Latvian students were found to have severe or extremely severe depression. The results regarding anxiety were significantly higher: 31.7% of students had severe or extremely severe anxiety, compared to 21.6% in the Saudi Arabian study [8]. The highest scores for depression, anxiety, and stress were confirmed for students from the Faculty of Veterinary Medicine. According to the conclusions of Pacheco et al. [40], medical students suffer from anxiety, depression, stress, and other mental health problems due to the high-pressure nature of their studies. To mitigate this negative impact, it is essential to provide students with opportunities for physical activity and encourage them to take full advantage of these opportunities. Engaging in physical activity helps manage stress and improve mental well-being [41].

The study data revealed that the most prevalent eating behavior among students was uncontrolled eating, indicating an uneven eating experience ranging from skipping meals to overeating. The study by de Lauzon et al. [42] also reported the highest average score for uncontrolled eating among young adults. This suggests that eating habits are influenced by emotions, which in turn are shaped by a combination of biological, psychological, and social factors. Meanwhile, the highest score for cognitive restraint was among students at the Poznan University of Medical Sciences, with males scoring 29.9 and females scoring 34.1 [11]. This study also confirmed a similar score regarding cognitive restraint, with an average score of 32.86. In addition, students from the Faculty of Agriculture and Food Technology had significantly (*p* < 0.05) higher cognitive restraint scores (38.55), which can be explained by the nutrition and food knowledge they acquired during their studies. Studies have shown that individuals with cognitive restraint eat more healthily and adhere more closely to the Mediterranean diet [11,43]. Students studying veterinary medicine showed the highest scores for uncontrolled eating and emotional eating compared to students from other faculties. This can be attributed to the six-year study period and increased practical work and responsibility involved in practicing in a university teaching clinic.

A strong positive correlation was found between uncontrolled eating and emotional eating among Latvian students. This finding is consistent with the results of a study of Portuguese university students, in which a positive correlation was also observed between eating behaviors such as emotional eating, rigid control, and binge eating [18]. Similar conclusions were also found in a study of the causes of emotional eating, in which emotional eating was associated with high rates of dietary restraint [44].

PSQI results indicated that LBTU students experienced poor sleep quality, with no significant differences observed between those from different fields of study (*p* > 0.05). Similar findings emerged for students at the University of Alicante, where the average PSQI score was 7.03 [22], and at Qazvin University of Medical Sciences, with an average PSQI score of 6.23 [45]. Analyzing the results regarding actual sleep duration over the past month, it was found that LBTU students slept an average of 6.59 h per night. The minimum and maximum recorded sleep durations were 4 and 12 h, respectively. Sleep duration of 4 h over a long period can significantly impact cognitive abilities and emotional balance [46], while excessive sleep (e.g., 12 h) is often associated with depression, burnout, and fatigue [47]. The average sleep duration of students is similar to that reported in other studies, for example, students at the University of Alicante slept for an average of 6.88 h [22], while students at a Vietnamese university slept for an average of 6.38 h [24]. The results of Li et al.’s [48] meta-analysis revealed that 52.3% of students get less than seven hours of sleep, which is insufficient according to the guidelines of the National Sleep Foundation.

Several significant relationships were confirmed when analyzing possible correlations between BMI, sleep quality, depression, anxiety, stress, uncontrolled eating, cognitive restraint, and emotional eating. BMI was found to have a weak but significant correlation with cognitive restraint (r = 0.266, *p* < 0.01) and emotional eating (r = 0.159, *p* < 0.01). The results provided an answer to the first research question, indicating that relationship exists and that further research involving a larger number of participants is needed. This finding is consistent with the results of Almuhammadi & Alfawaz’s [49] study, who observed a correlation between BMI and eating behavior, particularly cognitive restraint, in students. On other hand, a Dutch study found no link between emotional eating and BMI [50].

In response to the second research question, which concerned the relationship between mental health (depression, anxiety, and stress) and eating behavior (uncontrolled eating, cognitive restraint, and emotional eating), the results showed a very weak correlation between all three DASS-42 subscales (depression, anxiety, and stress) and the two eating behaviors (uncontrolled eating and emotional eating). This finding did not corroborate the results of Polish students, in which significant association was found between depressive symptoms and uncontrolled eating [11]. Similar findings were reported by Shafiq et al. [8], who found that depression and anxiety were associated with unhealthy eating habits among Pakistani students. A review by Solomou et al. [7] also discussed the link between mental health (depression, anxiety, and stress) and diet quality, concluding that a healthy diet ensures good mental health. In contrast, anxiety and stress lead to a poor diet. The results of the study indicated that students experiencing depression, anxiety, or stress were more prone to emotional eating. The data confirmed a weak but significant correlation between emotional eating and depression (r = 0.189, *p* < 0.01), anxiety (r = 0.182, *p* < 0.01), and stress (r = 0.193, *p* < 0.01). This means that students choose to eat not because they are hungry, but because they are experiencing positive or negative emotions.

Sleep quality was found to have a moderately strong correlation with depression (r = 0.508, *p* < 0.01), anxiety (r = 0.490, *p* < 0.01), and stress (r = 0.466, *p* < 0.01). This confirms the conclusion of Clement-Carbonell et al. [22], who found that sleep quality has a significant impact on mental health. The results supported the third research question, which was that sleep quality significantly impacts symptoms of depression, anxiety, and stress. The positive, strong correlation confirmed that this factor should be considered when devising preventive measures to enhance student well-being. However, Gürbüz & Bayram [51] also reported a moderately strong correlation between sleep quality and disordered eating. This finding was only partially confirmed in the present study, revealing a very weak correlation between sleep quality and uncontrolled eating (r = 0.102, *p* < 0.05) and emotional eating (r = 0.109, *p* < 0.05).

### Limitations of Study

The present study had its limitations. Among the limitations of the study, the number of students and their limited representation from different faculties should be noted. During the recruitment process, the researchers encountered a low response rate from students, particularly those from faculties such as the Faculty of Economics and Social Development and Faculty of Forest and Environmental Sciences. Various student groups on social media and WhatsApp were contacted in the hope of receiving a greater response. However, some students declined to participate in the study for various reasons. Another limitation of the study was the reliance on self-reported height and weight, which were used to calculate BMI. Although the accuracy of self-reported data cannot be guaranteed, the researchers considered deliberate misreporting by adult participants to be unlikely. A further limitation of this study was that student well-being was primarily measured using indicators such as mental health, sleep quality, eating behavior, and BMI, without taking into account other important factors such as physical activity, substance use, chronic health conditions and socioeconomic status. A limitation of the study lies in the data analysis methods relied primarily on the Pearson Spearman correlation test. Nevertheless, this study can be regarded as a pilot investigation to evaluate the need for further research. In this subsequent research, more robust approaches could be applied to a larger sample, such as regression models, mediation or moderation analyses, and structural equation modeling, to yield deeper insights. Furthermore, future research would benefit from adopting a longitudinal design to examine students’ mental health, sleep quality, eating habits, and BMI more comprehensively over time. This could involve taking repeated measurements at various points throughout the academic year (fall and spring semester), and at different stages of students’ academic progression (e.g., entry, mid-program, and completion of studies). This would provide a more comprehensive view of students’ mental health and potential solutions to improve it.

## 5. Conclusions

The study clearly showed that students were at high risk of depression, anxiety, and stress, which were linked to poor sleep quality and unhealthy eating behavior. These findings underscore the importance of regular mental health screening and support for university students. These results will inform the development of a support program designed to promote students’ mental well-being, which is essential for academic success.

## Figures and Tables

**Table 1:** The participants of the study.

Indicators		*n*	%
Gender	Female	264	66.5
	Male	130	32.7
	Other	3	0.8
The field of study	Faculty of Agriculture and Food Technology	66	16.6
	Faculty of Engineering and Information Technologies	98	24.7
	Faculty of Forest and Environmental Sciences	110	27.7
	Faculty of Economics and Social Development	64	16.1
	Faculty of Veterinary Medicine	59	14.9
BMI (total)	Underweight	27	6.8
	Healthy weight	238	59.9
	Overweight	85	21.4
	Obesity	47	11.8

**Table 2:** Depression, anxiety, and stress scores of students by field of study.

	Average Score ± SD	Min Value	Max Value
Depression			
Total	13.49 ± 10.10	0	42.00
Faculty of Agriculture and Food Technology	13.92 ± 10.53 ^a^	0	41.00
Faculty of Engineering and Information Technologies	14.27 ± 10.25 ^a^	0	42.00
Faculty of Forest and Environmental Sciences	12.49 ± 10.18 ^a^	0	42.00
Faculty of Economics and Social Development	12.47 ± 9.59 ^a^	1.00	40.00
Faculty of Veterinary Medicine	14.69 ± 9.83 ^a^	1.00	37.00
Anxiety			
Total	11.24 ± 8.90	0	39.00
Faculty of Agriculture and Food Technology	12.64 ± 9.67 ^a^	1.00	39.00
Faculty of Engineering and Information Technologies	10.88 ± 8.42 ^a,b^	0	35.00
Faculty of Forest and Environmental Sciences	9.73 ± 8.54 ^b^	0	39.00
Faculty of Economics and Social Development	11.44 ± 8.81 ^a^	1.00	38.00
Faculty of Veterinary Medicine	12.90 ± 9.26 ^a^	0	35.00
Stress			
Total	16.47 ± 9.96	0	42.00
Faculty of Agriculture and Food Technology	17.58 ± 10.19 ^a,b^	0	39.00
Faculty of Engineering and Information Technologies	15.33 ± 9.07 ^c^	0	35.00
Faculty of Forest and Environmental Sciences	12.49 ± 10.18 ^d^	0	42.00
Faculty of Economics and Social Development	17.05 ± 10.41 ^b,c^	3.00	41.00
Faculty of Veterinary Medicine	19.56 ± 10.09 ^a^	0	39.00

SD standard deviation. Significant differences (*p* < 0.05) within the column (between faculties) and subscale are indicated by different letters.

**Table 3:** Three Factors Eating Questionnaire and Pittsburgh Sleep Quality Index (PSQI) of students by field of studies.

	Average Score ± SD	Min Value	Max Value
Uncontrolled eating			
Total	43.72 ± 14.69	18.52	81.48
Faculty of Agriculture and Food Technology	43.94 ± 15.41 ^a^	18.52	77.78
Faculty of Engineering and Information Technologies	42.36 ± 15.34 ^a^	18.52	81.48
Faculty of Forest and Environmental Sciences	46.16 ± 13.43 ^a^	22.22	81.48
Faculty of Economics and Social Development	37.96 ± 10.60 ^b^	18.52	59.26
Faculty of Veterinary Medicine	47.39 ± 16.98 ^a^	22.22	77.78
Cognitive restraint			
Total	32.86 ± 30.58	0	100
Faculty of Agriculture and Food Technology	38.55 ± 32.45 ^a^	0	100
Faculty of Engineering and Information Technologies	31.06 ± 30.89 ^a^	0	100
Faculty of Forest and Environmental Sciences	32.22 ± 29.41 ^a^	0	100
Faculty of Economics and Social Development	30.90 ± 27.18 ^a^	0	88.89
Faculty of Veterinary Medicine	32.77 ± 31.68 ^a^	0	100
Emotional eating			
Total	31.14 ± 29.00	0	100
Faculty of Agriculture and Food Technology	35.01±31.68 ^a^	0	100
Faculty of Engineering and Information Technologies	30.84 ± 29.87 ^a^	0	100
Faculty of Forest and Environmental Sciences	30.40 ± 26.24 ^a^	0	94.44
Faculty of Economics and Social Development	19.53 ± 18.73 ^b^	0	77.78
Faculty of Veterinary Medicine	42.24 ± 32.80 ^a^	0	94.44
Pittsburgh Sleep Quality Index (PSQI)			
Total	11.01 ± 3.03	5.50	21.00
Faculty of Agriculture and Food Technology	10.53 ± 3.18 ^a^	6.00	21.00
Faculty of Engineering and Information Technologies	11.48 ± 3.13 ^a^	6.00	19.00
Faculty of Forest and Environmental Sciences	11.04 ± 2.84 ^a^	6.50	20.50
Faculty of Economics and Social Development	10.47 ± 2.87 ^a^	5.50	17.50
Faculty of Veterinary Medicine	11.32 ± 3.13 ^a^	6.50	21.00

SD standard deviation. Significant differences (*p* < 0.05) within the column (between faculties) and subscale are indicated by different letters.

**Table 4:** Correlations between BMI, PSQI, DASS-42, and R18V2.

		BMI	PSQI	DASS-42			R18V2		
				Depression	Anxiety	Stress	UE	CR	EE
BMI	Coefficient	-							
	*p*	-							
PSQI	Coefficient	0.039	-						
	*p*	>0.05	-						
Depression	Coefficient	0.027	0.508	-					
	*p*	>0.05	<0.01	-					
Anxiety	Coefficient	−0.105	0.490	0.642	-				
	*p*	<0.05	<0.01	<0.01	-				
Stress	Coefficient	−0.046	0.466	0.694	0.832	-			
	*p*	>0.05	<0.01	<0.01	<0.01	-			
UE	Coefficient	0.084	0.102	0.113	0.117	0.111	-		
	*p*	>0.05	<0.05	<0.05	<0.05	<0.05	-		
CR	Coefficient	0.266	0.034	0.077	0.066	0.092	0.280	-	
	*p*	<0.01	>0.05	>0.05	>0.05	>0.05	<0.01	-	
EE	Coefficient	0.159	0.109	0.189	0.182	0.193	0.636	0.315	-
	*p*	<0.01	<0.05	<0.01	<0.01	<0.01	<0.01	<0.01	-

UE—uncontrolled eating; CR—cognitive restraint; EE—emotional eating.

## Data Availability

The data are available on request from corresponding author.

## Abbreviations

The following abbreviations are used in this manuscript:

LBTU	Latvia University of Life Sciences and Technologies
BMI	Body mass index
DASS	Depression, anxiety, and stress scale
R18V2	Three-factor eating questionnaire
PSQI	Pittsburgh sleep quality index
